# What to consider for ECG in mice—with special emphasis on telemetry

**DOI:** 10.1007/s00335-023-09977-0

**Published:** 2023-02-07

**Authors:** Charlotte Calvet, Petra Seebeck

**Affiliations:** Zurich Integrative Rodent Physiology (ZIRP), University of Zurich, Zurich, Switzerland

## Abstract

Genetically or surgically altered mice are commonly used as models of human cardiovascular diseases. Electrocardiography (ECG) is the gold standard to assess cardiac electrophysiology as well as to identify cardiac phenotypes and responses to pharmacological and surgical interventions. A variety of methods are used for mouse ECG acquisition under diverse conditions, making it difficult to compare different results. Non-invasive techniques allow only short-term data acquisition and are prone to stress or anesthesia related changes in cardiac activity. Telemetry offers continuous long-term acquisition of ECG data in conscious freely moving mice in their home cage environment. Additionally, it allows acquiring data 24/7 during different activities, can be combined with different challenges and most telemetry systems collect additional physiological parameters simultaneously. However, telemetry transmitters require surgical implantation, the equipment for data acquisition is relatively expensive and analysis of the vast number of ECG data is challenging and time-consuming. This review highlights the limits of non-invasive methods with respect to telemetry. In particular, primary screening using non-invasive methods can give a first hint; however, subtle cardiac phenotypes might be masked or compensated due to anesthesia and stress during these procedures. In addition, we detail the key differences between the mouse and human ECG. It is crucial to consider these differences when analyzing ECG data in order to properly translate the insights gained from murine models to human conditions.

## Introduction

The mouse is the predominant mammalian model in experimental research. Genetically or surgically altered mice are commonly used as models of human cardiovascular diseases. The number of genetically engineered mice with cardiac phenotypes strongly increased during the last years and their cardiovascular physiology needs to be characterized before they are used for dedicated research questions (Birling et al. 2021; James et al. 1998; Svenson et al. 2003). Cardiac phenotyping requires reproducible, high-throughput methods, which should be conducted in an accurate and standardized manner making sure that findings are not missed, anticipated or biased (Ehlich et al. 2022; Hoit and Nadeau 2001; Hoit 2004). Many phenotypes are subtle, and mice are often able to compensate for mutations in order to maintain blood pressure and cardiac output within a healthy range (Kass et al. 1998). Additionally, pathological phenotypes might become more pronounced during aging (Merentie et al. 2015).

Non-invasive techniques like echocardiography and surface ECG recordings are easy and fast to perform and are therefore used for primary screening of the murine cardiovascular system during large-scale primary phenotyping pipelines (Hartley et al. 2002). The German mouse clinic as a member of the International Mouse Phenotyping Consortium (IMPC) has set up a practical guide for echocardiography and ECG for screening the cardiovascular system of one mouse in 10 min (Moreth et al. 2014).

Echocardiography has become standard to quantify murine heart physiology and function as well as to identify cardiac phenotypes and responses to pharmacological and surgical interventions (Hoit 2003; 2006; Schmidt et al. 2002; Syed et al. 2005). Besides heart anatomy, it provides information on the mechanical function of heart muscle, vessels and valves as well as blood flow patterns; however, echocardiography does not provide any information about the electrical activity of the murine heart’s conduction system.

Electrocardiography (ECG) is the gold standard for assessing cardiac electrophysiology. ECG differences have been reported between different mouse lines and during development and aging (Goldbarg et al. 1968; Heier et al. 2010; Moreth et al. 2014). Unfortunately, a variety of methods are used for mouse ECG under diverse conditions, making it difficult to compare different results (Ho et al. 2011; Wehrens et al. 2000). Additionally, non-invasive techniques require the mouse to be taken out of its home cage, restrained or even anaesthetized. It is known that stress, pain and anesthesia strongly influence cardiovascular as well as other physiological parameters, so that these data cannot be taken as unaffected normal values and must be interpreted with caution (Arras et al. 2007; Cinelli et al. 2007; Gaburro et al. 2011; Taitt and Kendall 2019). Additionally, with non-invasive techniques no long-term data can be acquired, which is of relevance since cardiovascular and other diseases progress with aging and hence affect the ECG pattern (Goldbarg et al. 1968; Heier et al. 2010; Kim et al. 2022; Merentie et al. 2015; Moreth et al. 2014; Stables et al. 2016).

Implantable telemetry devices allow continuous ECG recording over a period of up to several months in freely moving conscious and undisturbed mice (Kramer et al. 1993; Cesarovic et al. 2011). Telemetry offers the acquisition of continuous ECG data 24/7 with mice in their home cage environment and therefore allows the acquisition of unaffected normal values (Arras et al. 2007; Cesarovic et al. 2011). Additionally, most telemetry systems collect additional physiological parameters (e.g. activity, blood pressure and body temperature) simultaneously and telemetry could be combined with specific challenges, e.g. surgical or pharmacological interventions, feeding or behavioral tests (Gaburro et al. 2011).

Mouse models are used to study pathophysiological mechanisms of human cardiovascular diseases, nevertheless, there are some differences between the mouse and human ECG—which should be well-known and considered during analysis, in order to allow the insights gained from murine models to be properly translated to human conditions (Boukens et al. 2014; Kaese and Verheule 2012).

The aim of this review is to give details on the mouse ECG, discuss its difference to human ECG and give examples of arrhythmias. Furthermore, we compare the different methods used to record ECG in mice and provide information on mouse ECG telemetry, its pros and cons, what has to be considered when using it and what information can be gained from it.

## The mouse ECG

In 1968, Goldbarg et al. published for the first time a mouse ECG description (Goldbarg et al. 1968). Since then, the electrocardiogram (ECG) has become the “gold standard” for the analysis of cardiac electrophysiology. Mouse models are widely used in cardiovascular research despite noticeable differences with humans such as the small size, fast heart rate (HR), and differences in surface ECG. Reliable and repeatable measurements of mouse ECG are of great importance for the translation of results to human diseases (Kramer et al. 1993; Merentie et al. 2015).

Therefore, this paragraph aims to give a brief overview on the mouse ECG, its differences to the human ECG, common types of arrhythmias and factors influencing the cardiac activity and thus ECG in mice.

### ECG differences between mouse and human

**Fig. 1:**
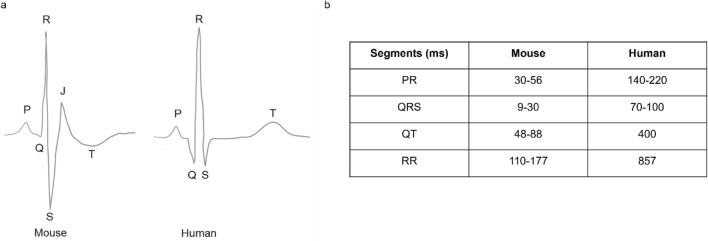
ECG and interval differences between mouse and human. **a** A typical mouse ECG compared to human ECG. The different waves (P, Q, R, S, J, T) are represented and are distinct in mouse and human. Note that a small J wave might be visible in human depending on lead placement. **b** Mouse and human ECG intervals in milliseconds

The mammalian heart functions as a pump, its contraction occurs after the generation of an action potential and its propagation, followed by a relaxation period and a refraction period before the generation of the next impulse (Nerbonne and Kass 2005). Electrical activity in the myocardium is manifesting on the surface ECG, revealing the current propagation through the atria (P wave) to the depolarization (QRS complex), followed by the repolarization of the ventricles (T wave) (Nerbonne 2014) (Fig. 1). Depending on lead placement, the ECG has different shapes (Tremoleda et al. 2012). In mice, the lead II derivation—corresponding to the right arm to the left leg axis—is generally used because it results in the strongest signal (Steijns et al. 2020). The HR of mice (450–725 beats/min) is ten times higher than the HR of humans (60–100 beats/min) (Kramer et al. 1993; Janssen et al. 2002; Kaese and Verheule 2012). Compared to humans (150–380 g), the mouse heart weight (around 200 mg) is proportionally reduced in size (Doevendans et al. 1998; Schüttler et al. 2020).

The duration and waveforms of action potentials in mouse and human atrial and ventricular myocytes are different (Schüttler et al. 2020). In the mouse, the repolarization is rapid so that no plateau phase can be observed as in human during repolarization (Nerbonne 2014; Schüttler et al. 2020; Tomsits et al. 2021). Because of these differences, the mouse ECG reveals significantly shorter intervals than the human (Doevendans et al. 1998; Kaese and Verheule 2012; Puckelwartz et al. 2009; Gottlieb et al. 2021) (Fig. 1).

The mouse P wave is similar to the human P wave and corresponds to atrial activation. In humans, the non-isoelectric PR segment represents atrial repolarization (Holmqvist et al. 2009; Boukens et al. 2014) (Fig. 1). Compared to most mammals, the morphology of the QRS complex is different in mice, the S wave is immediately followed by a distinct J wave (Goldbarg et al. 1968; Doevendans et al. 1998; Boukens et al. 2013). This J wave is associated to early repolarization and linked to the high heart rate in this species (Offerhaus et al. 2021). The J wave is slightly visible in humans (Kaese and Verheule 2012). Due to the presence of the J wave in mice, the end of the QRS complex is not clearly distinguishable. Therefore, in mice, the QRS complex is including ventricular activation as well as early repolarization (Boukens et al. 2013) (Fig. 1). The presence of an isoelectric ST segment is not clear in mice as the T wave amplitude is relatively small (due to the absence of a plateau phase) and merges with the end of the QRS complex (Goldbarg et al. 1968; Berul et al. 1996; Doevendans et al. 1998; Boukens et al. 2013). The end of the T wave in mice mirrors the end of ventricular repolarization (Boukens et al. 2013; London 2001) (Fig. 1).

### Factors influencing ECG

ECG differences have been reported between different mouse strains and sexes and during development and aging (Goldbarg et al. 1968; Heier et al. 2010; Howden et al. 2008; Merentie et al. 2015; Moreth et al. 2014; Campen et al. 2005). Strain-specific differences have been reported for heart dimensions, cardiac structure and function, affecting the mouse ECG e.g. in terms of HR and heart rate variability (HRV—time variation from beat to beat) (Howden et al. 2008; Moreth et al. 2014). As in humans (Prajapati et al. 2022), different studies reported sex differences in mice, for example during cardiac repolarization—the K^+^ current density is lower in female ventricular myocytes (Trépanier-Boulay et al. 2001), this difference might be explained by sex-specific hormones (Saito et al. 2009; Brouillette et al. 2005). Female mice seem to be more susceptible to hyperoxia (e.g. in terms of severe bradycardia, PR shortening and QTc prolongation, mortality) and unlike in male mice, no hypertrophy upon hyperoxia exposure has been observed in female mice (Rodgers et al. 2019). Small differences were also observed in ECG interval durations between female and male mice (Moreth et al. 2014). Also, different drug-related ECG-changes have been observed in males and females (Warhol et al. 2021).

Age is also playing an important role in mouse cardiac function. HR, duration of P wave, and PQ interval have been shown to increase with age (Merentie et al. 2015; Jansen et al. 2021). In addition, Merentie et al. identified a decrease in the R-wave amplitude with age (Merentie et al. 2015). The same authors reported an increase of the prevalence of arrhythmias, such as premature atrial contractions (PACs) or atrial fibrillation with age (Merentie et al. 2015; Jansen et al. 2021). These results mirror well what is observed in humans as the aging human heart is more prone to arrhythmias (Mirza et al. 2012). Another study reported that HRV(is reduced in aging mice and strongly correlated with the frailty index reflecting the health status of the animal (Dorey et al. 2021).

Cardiac activity has a marked diurnal pattern and strongly depends on the animal’s activity/sleep–wake rhythm with higher HR during the active (night) phase. Mice with a disruption of the circadian clock have shown an increased vulnerability to arrhythmias (Gottlieb et al. 2021). Different studies demonstrated that environmental temperature impacts cardiac activity, mice housed at lower temperatures have higher HRs (Axsom et al. 2020; Chan et al. 2019; Farah et al. 2004). In contrast, mice in torpor showed a significantly decreased HR in combination with lowered body temperature (Swoap and Gutilla 2009). Similarly to larger species and humans, mice also have a significantly increased HR during exercise compared to resting (Desai et al. 1997; Lujan et al. 2012).

Anesthesia, pain or chronic diseases also influence the HR (Arras et al. 2007; Ho et al. 2011; Stables et al. 2016; Stypmann 2007; Kim et al. 2022; Yang et al. 1999). Additionally, a decrease in HRV can be observed in mice in response to physiological changes (e.g. in environment or with age) (Fenske et al. 2016; Kovoor et al. 2001; Ho et al. 2011; Dorey et al. 2021).

Anesthesia can induce significant changes in systemic physiology and introduce research bias. On the one hand, most of the anesthetics are known to have a depressive effect on HR. On the other hand, comparing data between studies can be complicated due to the use of different anesthesia protocols (Ho et al. 2011; Wehrens et al. 2000). Therefore, ECG recordings in conscious animals are ideal when possible (Kurtz et al. 2005). If anesthesia must be used for ECG recordings an anesthetic agent with the least effect on HR suppression must be used (Yang et al. 1999; Stypmann 2007; Ho et al. 2011). For example, inhaled halogenated ethers (e.g. isoflurane) have rather small effects on the mouse ECG (Chaves et al. 2003; Warhol et al. 2021). On the contrary, ketamine/xylazine mixture have a pronounced effect on HR depression and should be avoided if possible (Hart et al. 2001; Kawahara et al. 2005; C. Lee and Jones 2018). Additionally, another study reported that anesthesia may mask a cardiac phenotype (e.g. left ventricular dysfunction) and cardiac diseases may therefore be better evaluated in conscious mice (Lairez et al. 2013).

Pain and disease progression have been shown to influence HR and HRV. Several studies revealed that post-surgical or chronic pain increases HR in combination with a decrease in HRV in mice (Arras et al. 2007; Stables et al. 2016; Kim et al. 2022) as in humans (Faye et al. 2010; Hallman et al. 2011). Mice subjected to laparotomy with no pain medication exhibited an increased HR and decreased HRV. Other physiological parameters have also been affected such as an increase of core body temperature and decrease of body weight (Arras et al. 2007). As in humans, different diseases (e.g. epilepsy, diabetes) have been shown to affect ECG in mice. A study revealed that mice with epilepsy were prone to arrhythmias and had a high risk of sudden cardiac arrest (Mishra et al. 2018) and another publication reported that diabetes type 1 decreases HR in mice (Lin et al. 2010). Mice developing peritoneal metastasis after an intraperitoneal injection of mouse colon carcinoma cells showed a decrease in HRV (Kim et al. 2022). Therefore, HR and HRV assessment seems to be a useful tool for evaluating pain and disease progression.

Finally, mice are prone to stress in particular when handled, restrained or put into an unfamiliar environment. Different studies have shown that scruffing them increases their HR in combination with a reduction in HRV (Cinelli et al. 2007; Taitt and Kendall 2019). Kramer et al. revealed that mouse HR was increased to 700–750 beats/min during weighing procedure and up to 800 beats/min during handling or after cage change compared to a normal HR during resting period of 450–500 beats/min (Kramer et al. 1993). In contrast, if firmly restrained they might even exhibit bradyarrhythmia—presumably due to pressure on cervical baroreceptors during scruffing the neck skin (Labitt et al. 2021). Restraining healthy mice has also been shown to induce severe arrhythmias (e.g. second- and third-degree atrio-ventricular blocks) and sinus pauses (moments without atrial activity) a few minutes after releasing the restraint (Labitt et al. 2021). Social stress, such as single housing, is associated to an increase in HR compared with mice housed in pairs (Späni et al. 2003). HR and HRV have been also used to assess individual differences in fear response magnitudes. More anxious mouse lines were found to have higher HRs when exposed to new environments (Gaburro et al. 2011; D. L. Lee et al. 2004; Liu et al. 2014; Sgoifo et al. 2014). Thus, the determination of a baseline HR and HRV is important to characterize new phenotypes (Ho et al. 2011).

### Arrhythmias

Mice are valuable models in uncovering new mechanisms implicated in human cardiac diseases and ECG is the method of choice to detect arrhythmias (Dobrev and Wehrens 2018). Several factors mentioned before can cause arrhythmias (e.g. stress, age, epilepsy) (Labitt et al. 2021; Mishra et al. 2018; Merentie et al. 2015). An arrhythmia is characterized by an irregular heart beat rhythm, it could be too slow (bradycardia), too fast (tachycardia) or characterized by the presence of extra-beats (Anwar et al. 2018). Arrhythmias can be divided into supra-ventricular arrhythmias, ventricular arrhythmias or atrio-ventricular (AV) conduction disturbance, also called AV blocks (Table 1).

**Table 1:** Most common arrhythmias are classified according to their origin

Supra-ventricular	Bradycardia	Sinus node dysfunction
	Tachycardia	Atrial fibrillation Atrial futter Paroxysmal supra-ventricular tachycardia
	Extra-beats	Premature atrial contraction (PAC) Premature junctional contraction (PJC) Atrial ectopic beats
Ventricular	Tachycardia	Ventricular fibrillation Ventricular tachycardia
	Extra-beats	Premature ventricular contraction (PVC) Ventricular ectopic beats
AV blocks	Bradycardia	First-degree
		Second-degree
		Third-degree

Atrial fibrillation is a supra-ventricular arrhythmia and the most common cardiac arrhythmia in humans (Wyndham 2000). In both mice and humans it manifests by an irregular and often abnormally fast HR with no visible P waves and an irregular QRS complex (Schüttler et al. 2020; Herrmann et al. 2011) (Table 2). Atrial flutter is characterized by a fast atrial rate with a fixed or variable ventricular rate, ECG recordings show a sawtooth pattern without an isoelectric line in between QRS complex (Cosio 2017; Ziccardi et al. 2022) (Table 2). Paroxysmal supra-ventricular tachycardia is defined by intermittent episodes of tachycardia at rest (Hafeez et al. 2022). Premature atrial contractions (PAC) when isolated are most often benign findings. PAC are contractions of the atria not originating from the sinoatrial node. On ECG recordings, the RR interval is shortened relative to the previous cycle, and the P wave typically occurs earlier than usually and its shape is often modified (Sgoifo et al. 2014; Heaton and Yandrapalli 2022) (Table 2).

**Table 2:**
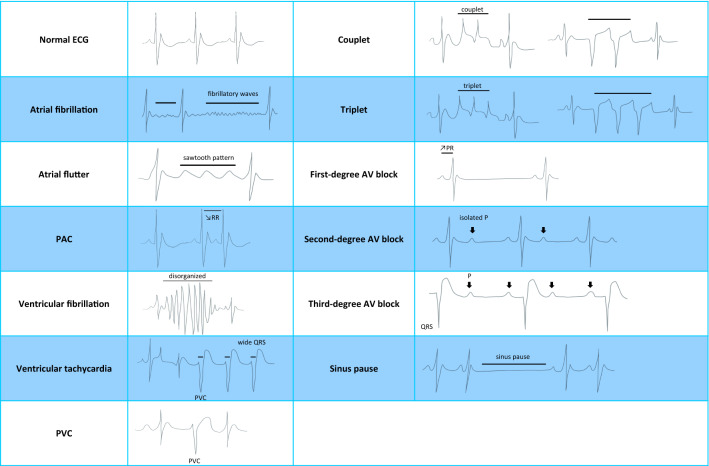
Different types of arrhythmias in mice and representative ECG graphics

As part of the ventricular arrhythmias, ventricular fibrillation is causing a typical rapid and disorganized rhythm of heart beats and can lead to sudden cardiac death (Ludhwani et al. 2022; Herrmann et al. 2011) (Table 2). Ventricular tachycardia is characterized by a fast abnormal HR with a wide QRS complex (Foth et al. 2022; Sgoifo et al. 2014) (Table 2). Premature ventricular complex (PVC) is a single premature ventricular ectopic beat bracketed by two sinus (non-ventricular) beats (Table 2). Two consecutive PVCs are termed doublets while three consecutive PVCs are named triplets and they can manifest in different shapes (Table 2) (Farzam and Richards 2022; Sgoifo et al. 2014).

Bradyarrhythmias are due to sinus node dysfunction (SND) or atrio-ventricular (AV) conduction disturbances also named blocks (Vogler et al. 2012). An AV block represents a delay in the transmission of an electrical impulse from the atria to the ventricles and is characterized by a slow HR (Kashou et al. 2022) (Table 2). A first-degree AV block is defined as a prolongation of PR interval and the P waves always precede the QRS complexes (Kashou et al. 2022). In a second-degree AV block there is a significantly elongated PR interval due to an intermittent atrial to ventricle conduction until eventually an atrial impulse is completely blocked, resulting in an isolated non-conducting P wave (Kashou et al. 2022; Mangi et al. 2022). A third-degree AV block is characterized by an absence of AV nodal conduction, and the P waves are never related to the QRS complexes (Kashou et al. 2022; Nakamura et al. 2001).

## Non-invasive methods for ECG acquisition

Most non-invasive methods for ECG acquisition are based on detecting the cardiac signal through the plantar surfaces of the animal’s paws. This can be either achieved by placing the awake freely moving or restrained animal on a small, instrumented platform or the anesthetized animal on a platform with its limbs fixed to defined small, instrumented areas (Table 4).

Both, the freely moving and the restrained system can be used in an easy and high-throughput manner (Moreth et al. 2014). Since these systems requires no anesthesia or restraint, they are especially valuable for fragile transgenic and knockout animals as well as newborn pups (Kumstel et al. 2020). However, for the systems where animals have to be taken out of their home cage environment (and restrained) the mice are assumed to be under a certain stress. Additionally, all of these systems are most likely to be used during people’s normal working hours (which is the resting phase of mice) and acquisition during night (the active phase of mice) is more challenging. Newly developed non-invasive telemetry jackets are available but so far only for larger animals, rats and hamsters (Fares et al. 2022; Flenet et al. 2020).

### Paw surface recording in the awake freely moving mouse

The animal is placed on a small, instrumented platform (e.g. ECGenie from Mouse specifics, Inc., Framingham, MA, USA) and cardiac electrical activity is detected through the animal’s paws. After an acclimatization period, ECG can be acquired for several minutes in freely moving mice and neonatal mice (ECGenie); a multi-platform system allows collecting ECG data from multiple mice in parallel. However, movement impacts ECG data quality.

A multi-dry-electrode plate (MDEP) sensor (Unique Medical, Tokyo, Japan) has been developed to monitor ECG and HR in freely behaving mice. This system can be placed at the bottom of the home cage to monitor mice in their environment. It contains 15 electrodes and ECG signals are detected when at least two paws of a mouse are in contact with two different electrodes (Sato 2019).

### Paw surface recording in the awake restrained mouse

This methodology is based on a restraining system (EasyCG®, EMKA technologies, France). The four sensors of the system platform, one for each paw, have to be coated with conductance gel, and the animals are placed on the platform and covered with a translucent half-tunnel of the appropriate size. The four wires of the platform are connected to a wireless transmitter and amplifier system (Mongue-Din et al. 2007). Compared to ECG acquisition in freely moving mice, the restrained system allows screenings with reduced movement artifacts.

### Paw surface recording in the anesthetized mouse

The anesthetized mouse is placed on a platform with its paws attached to three paw sized electrodes (e.g. Rodent Surgical Monitor + , Indus Instruments, Webster, TX, USA; small animal physiological monitoring system, Harvard apparatus, Holliston, MA, USA). Conductance gel is applied to improve conductivity. These systems contain a warming system and are commonly used to monitor animals during surgeries or imaging procedures and can be also used on pups (Kulandavelu et al. 2006). Most often, additionally to ECG, these systems also provide real-time information on other physiological parameters (e.g. body temperature, SpO_2_, blood pressure and respiration). Since the animal is anesthetized, accurate ECG data can be acquired without movement artefacts. However, all anesthetics are known to affect the cardiovascular system with different agents having different effects. Additionally, these platforms allow ECG acquisition for only one animal at the time, so their use for ECG analysis in larger cohorts of mice is time-consuming.

### Telemetry jackets for ECG acquisition in freely moving animals

Telemetry jackets are available for larger animals since a long time, but not for rodents so far. Recently, a company developed a non-invasive cardiorespiratory jacket to externally monitor ECG, respiratory function and activity level (Etisense, Lyon, France). So far, this system is currently only available for rats and hamsters. These jackets seem to perform similar to implanted telemetry in terms of heart rate detection (Fares et al. 2022; Flenet et al. 2020).

## Invasive methods

Invasive methods for ECG acquisition are based on detecting the cardiac signal through ECG leads which are placed subcutaneously. The leads are either connected to an external recorder in combination with a tether system or to a transmitter body (which is either implanted subcutaneously or intraperitoneally), transmitting data to an external recorder in a wireless manner.

### Tethered ECG systems

Tethered systems include four electrodes, which are tunneled under the skin towards the four limbs and bundled to exit via a mid-scapular incision where they are fixed on the back of the animal. The wires are connected to an external recorder via a tether system (a harness or jacket in larger animals) and a swivel arm. This allows the animal a certain degree of freedom and comfortable environment. However, surgical implantation is required and most mice do not tolerate harnesses very well. The externalized wires on the animal’s back are prone to wound infections and manipulation by the animal. Additionally, mice with externalized implants are often housed singly (Steijns et al. 2020).

### Telemetry systems

Telemetry offers continuous long-term acquisition of ECG data in conscious freely moving mice in their home cage environment and therefore allows the acquisition of unaffected normal values (Arras et al. 2007; Cesarovic et al. 2011). The first mouse telemetry system has been reported 50 years ago, since then the systems have been continuously improved in terms of transmitter size and biocompatibility as well as quality of data acquisition (Carson et al. 1972). Nowadays, miniaturized transmitters are available for mice, requiring surgical implantation. Transmitter body and wires are completely internalized, thus minimalizing the risk of damage and infection and allowing chronic long-term implantation of ECG transmitters (Cesarovic et al. 2011; McCauley et al. 2010; Fenske et al. 2016). Depending on the system (transmitter with or without battery, transmitter’s battery life) and schedule (acquisition rate, continuous or scheduled data acquisition) (Table 3) used, ECG data can be acquired over a period of up to several months (Kramer et al. 1993; Cesarovic et al. 2011). Telemetry could also be combined with specific challenges, e.g. surgical or pharmacological interventions, feeding or behavioral tests (Gaburro et al. 2011). However, mice implanted with transmitters cannot be used with certain methods e.g. micro CT or MRI, due to containing metal parts. Additionally, most telemetry systems collect other physiological parameters (e.g. activity, blood pressure and body temperature) simultaneously, adding valuable additional information.

**Table 3:** Technical specifications of transmitters from different providers

Company	DSI	Stellar telemetry, TSE		ADInstruments/Kaha sciences	Emka	Starr-Oddi
Country	USA	USA		New Zealand	France	Iceland
Model	ETA-F10	XS		MT10B	EasyTEL S-ETA	DST micro-HRT
		Memory	Real-time			
Weight (gram)	1.6	3.9	4.2	3.5	3.3	3.3
Volume (cc)	1.1	2.4	1.9	1.8	1.8	2.1
Battery life (days)^a^	60	90	180	No battery*	90	90
Acquisition rate	1000 Hz	100/200/ 500/1000 Hz	1/100/ 250/500 Hz	2 kHz	500 Hz	100–800 Hz
Transmitters/receiver unit	1	Up to 8	> 100	Up to 40	1	Up to 10
Transmission range	30 to 45 cm	5 m	3 m	7 cm	Few cm	20–30 cm

*Receives power from inductive wireless power transfer

^a^Continuous recording

This paragraph aims to give a brief overview on the common features and differences of telemetry systems. All systems are based on a combination of transmitters, a receiver, most often amplifier and a computer for data collection. A dedicated software for data acquisition and analysis is offered by each company.

#### Types of transmitters and hardware setup for data acquisition

Independently of the system used, the implant size must be adapted to the animal model and several telemetry systems are commercially available to monitor ECG in mice. The systems differ in their specifications (Table 3), but share the following components:

1/the transmitter containing a body (often recording also temperature and/or activity) and two ECG leads (except Starr-Oddi (Iceland) offering a leadless device to measure HR, activity, and temperature)—which can be combined with additional biopotential leads (e.g. for EEG or EMG) or a pressure sensor. Data are collected in real-time via a receiver. Most transmitters are not capable of storing data, except the ones from Starr-Oddi and Stellar Telemetry providing memory implants to remotely record data besides the ones for real-time data acquisition. Kaha sciences (now ADInstruments) transmitters do not contain a battery but receive power from an inductive wireless power transfer which has to be placed close to the cage. A recent review about the reporting quality in mouse ECG telemetry studies showed that the most frequently used models were ETA-F20 and ETA-F10 (with the ETA-F10 being the successor version of the ETA-F20) from DSI (Gkrouzoudi et al. 2022).

2/the receiver is specifically collecting one or multiple frequencies depending on the implant (one-channel or more leads) and system used. The animals’ home cages can be either placed on top of receiver plates (allowing to collect only data from one transmitter at the same time) or the receiver is placed in a certain proximity to the transmitters, allowing to monitor different animals at the same time. The Stellar memory implants remotely record data away from the receiver and are transmitted whenever the implant is within the range of the receiver, the Starr-Oddi transmitter data need to be extracted from the transmitter after its recovery. A metal shielding is recommended to isolate the system from electromagnetic interferences.

3/an acquisition computer and analysis software.

For real-time data acquisition, it is necessary to create a dedicated network to permit the hardware to communicate to the data acquisition computer. After the transmission of the data from the implant to the receiver, the data is collected using a data acquisition software on a computer. Data acquisition requires a dedicated mostly system-specific software provided with the system.

#### Surgical implantation of ECG telemetry transmitters

For the implantation of telemetry devices previous experience with mouse surgical procedures as well as training on dead animals is recommended. Preferably, gas anesthesia is used for anesthetizing the animal during surgery, because of its easy adjustment and fast wake up time after surgery. Analgesia needs to be provided peri- and postoperatively. Additionally, state of the art peri- and postoperative care including warming, fluid supply, intensive care nutrition, should be provided during the recovery period (Hankenson et al. 2018; Skorupski et al. 2017).

Typically, the surgical procedure for transmitters without ECG leads and suture ribs will require around 10–20 min and transmitters with ECG leads and suture ribs will require around 30–40 min of surgical anesthesia. Protocols and video tutorials for transmitter implantation are available from the vendors or published (Cesarovic et al. 2011; McCauley et al. 2010; Fenske et al. 2016).

The surgical area should be adequately clipped and any hair remnants should be removed carefully. Eye ointment should be applied and the animal placed on a warming pad in dorsal recumbency, the animal’s legs can be loosely taped to the warming pad (using medical tape) to avoid unwanted movement during surgical manipulation. The surgical area should be sufficiently disinfected and draped (using sterile drapes).

It is recommended to use a microscope for magnification and dedicated (micro-surgical) instruments of an appropriate size. Aseptic surgical technique should be used and all instruments, consumables and devices should be sterile.

Implants without any leads can be placed freely floating in the abdominal cavity. A small incision (of the size of the transmitter’s smallest diameter) is created in the midline (linea alba) of the ventral abdominal wall, whereas the skin and the abdominal wall are opened separately. The transmitter is slid into the abdominal cavity and the abdominal wall and skin are closed with separate 5–0 to 6–0 sutures.

Implants with ECG leads should be used with suture ribs when placed into the abdominal cavity and without suture ribs when placed subcutaneously (Cesarovic et al. 2011). If the collection of body temperature is required, the device should be placed within the abdominal cavity in order to measure body core temperature.

Suture ribs attached to the device body allow sutures to be threaded through them when closing the abdominal wall and thus to secure the transmitter body in the abdomen. Fixing it to the abdominal wall avoids putting tension on the ECG leads by migration of the transmitter body.

The intraperitoneal insertion of the transmitter body is identical to the procedure for implants without leads described above, the transmitter body should be placed on top of the intestines, parallel to the long axis of the body with the ECG leads oriented either cranially or caudally. The ECG leads are externalized by passing a larger (e.g. 18G) needle through the abdominal wall near the left and right (cranial or caudal depending on transmitter body orientation) lateral aspect of the incision and guiding the leads outside the abdominal cavity through the needle. The leads will be tunneled subcutaneously to the desired ECG electrode locations. If necessary, the lead material might be shortened to the appropriate length (take growth of the animal and re-use of the transmitter into account). The stainless-steel wire needs to be exposed at the tips of the ECG leads and it is recommended to fix them to the surrounding muscles. Finally, all wounds are closed as described above.

Transmitter bodies without suture ribs can be placed subcutaneously, either ventrally or dorsally via a midline skin incision. After opening the skin, a subcutaneous pouch is formed by blunt dissection, large enough to place the transmitter body inside without skin tension. The leads are tunneled subcutaneously to the desired ECG electrode locations and processed as described above for the intraperitoneal placement and all skin are finally closed.

After transmitter implantation mice should be given at least 5–7 days for recovery, recovery time can vary depending on age, sex, strain, genetic modifications, previous treatments or additional diseases.

## Data extraction and analysis

Analysis of ECG data is challenging due to the large amount of data acquired—this is especially relevant for telemetry data. One day of continuous recording will generate more than one million QRS complexes and three months recordings (corresponding to the average battery life) up to 100 million QRS complexes (Tomsits et al. 2021). This means that a pragmatic approach must be adopted to acquire, handle, analyze and interpret data. High quality data must be acquired to facilitate data analysis which mainly depend on the quality of transmitter implantation and lead positioning as well as the transmitter’s acquisition rate. Further, to minimize noise into the ECG signal during acquisition, implanted animals must be placed in a silent environment, shielding can be used to reduce noise interferences. Obtaining baseline ECG data from a continuous recording is recommended before performing any experiment, this would correspond to a minimal analysis of 20 consecutive QRS complexes in mouse (Tomsits et al. 2021). Due to a circadian alteration in ECG parameters in rodents as in humans, it is recommended to record light and night baselines (Tomsits et al. 2021).

The analysis of basic ECG parameters (e.g. HR, P wave duration, PR interval, QRS interval or QT duration), can be performed using system-specific software provided with the telemetry system (Table 3). These softwares allow to perform measurements, editing of data, performing calculations of derived parameters (e.g. average HR) and mathematical transformations (e.g. periodogram, fast Fourier transform). Manuals and videos explaining how to perform the analysis are provided with the telemetry system.

The softwares are equipped with automated analysis algorithms to identify P, Q and T waves. More precisely, templates are used for automated pattern recognition analysis. They correspond to predefined ECG cycles with accurately placed marks and can be matched to the entire ECG recording. HRV and changes (e.g. due to stress) might influence the results obtained with the automated analysis but specific periods can be reanalyzed using different thresholds. To facilitate analysis, filters can be applied to the data to remove noise or artefacts. Some research groups prefer to develop their own algorithms to detect ECG waves and specific patterns. After exporting raw data, digital signal processing can be performed using Matlab software (MathWorks, Natick, MA) (Böning et al. 2013; Fenske et al. 2016; Merentie et al. 2015; Thephinlap et al. 2011; Steijns et al. 2020).

The options used for data acquisition and analysis depend on the individual research project. Assessing basic ECG parameters with a lower acquisition rate and a scheduled data acquisition might be enough when characterizing a transgenic mouse model or investigating the effects of novel drug candidates in a disease model. A more detailed examination in combination with higher acquisition rate and continuous data acquisition of the ECG is necessary to detect specific patterns such as arrythmias. In this case, high quality data is necessary to properly annotate P and R waves and the pattern must be known and detected by the analyzer himself, which requires a specific knowledge (Merentie et al. 2015). Additional analysis modules are available to help for the detection of specific patterns. However, one should be aware that noise often masks small amplitude waves (e.g. P or T waves) and thus makes it difficult to automatically detect specific patterns (Hossain et al. 2019). At the end of the analysis process, recordings must be manually reviewed after the automated analysis to adjust individual data and add missing validation marks. The results can be saved and exported in different formats (e.g. raw data or excel files) for statistical analysis.

## Discussion

ECG is the gold standard for assessing cardiac electrophysiology. Besides HR it offers the analysis of several additional parameters, based on abnormalities of heart rhythm and regularity.

ECG data acquired with non-invasive techniques and telemetry and their analysis are identical, however, utilizing non-invasive techniques means that ECG is most often collected during the day which is the mice’s resting time. Additionally, mice as prey animals are stressed when handled, restrained or put into an unfamiliar environment (Cinelli et al. 2007; Labitt et al. 2021; Taitt and Kendall 2019). Thus, conscious mice analyzed with non-invasive techniques are supposed to show HRs beyond their resting levels (tachycardia) with a lower HRV. Subtle cardiac abnormalities might be masked or compensated due to stress during these procedures (Kass et al. 1998; Cinelli et al. 2007; Taitt and Kendall 2019). In contrast, when anesthetized mice are analyzed, it has to be considered that all anesthetics strongly influence cardiac parameters and thus might hamper the detection of a pathophysiological cardiac phenotype as well. Therefore, the acquisition of resting normal values should take place in conscious mice unaffected by stress during both activity and sleep periods. Phenotypic differences as a response to stress should be tested by specific standardized challenges, e.g. exercise, pharmaceutical or surgical interventions such as coronary artery ligation and transverse aortic constriction (Choy et al. 2016; Desai et al. 1997; Tarnavski 2009; Tomsits et al. 2021).

Arrhythmias might be subtle or absent in young animals or during the resting phase and they might develop and become more obvious during aging or the development of diseases (Goldbarg et al. 1968; Heier et al. 2010; Kim et al. 2022; Moreth et al. 2014; Stables et al. 2016). Additionally, cardiac activity has a marked diurnal pattern and strongly depends on activity/sleep–wake rhythm, environmental temperature, stress and pain—to name but a few.

Compared to non-invasive techniques, telemetry systems offer the benefit of continuous long-term recordings of ECG in conscious mice in their home cage environment. Additionally, telemetry offers to acquire data during different activities and sleep–wake rhythm (most relevant also during night which is the active phase of mice) and can be combined with different challenges like exercise, surgical or pharmacological interventions. Telemetry systems often provide additional parameters measured simultaneously e.g. activity, body core temperature or blood pressure which help to assess the animal’s activity pattern. Depending on the acquisition type and the transmitter’s battery life telemetry allows continuous ECG recording over a period of up to several months so they can be also used for ECG acquisition during aging or disease progression.

However, telemetry transmitters require surgical implantation and although mouse transmitters are already quite small and their size constantly decreases, they are they still relatively large and heavy for smaller mice (Table 4) and especially for fragile transgenic and knockout animals or mice which are pre-handicapped by a disease. Moreover, they cannot be used in pups. Additionally, mice implanted with transmitters cannot be used with certain methods e.g. micro CT or MR imaging, since they contain metal parts leading to imaging artefacts.

**Table 4:** Advantages and disadvantages of ECG monitoring methods

	Advantages	Disadvantages
Non-invasive ECG in freely moving mice	Non-invasive monitoring, screening of a large number of animals, conscious mice freely moving mice	Not possible for long-term monitoring, need adaptation, stressful, signal affected by movements
Non-invasive ECG in restrained mice	Non-invasive monitoring, better detection (no movement artefacts) conscious mice	Not possible for long-term monitoring, need adaptation, stressful,
Non-invasive ECG devices requiring anesthesia	Non-invasive monitoring, well adapted for monitoring during surgeries, monitoring of several parameters	Anesthesia needed, not possible for long-term monitoring, recording of only one animal at a time
Tethered systems	Intermediate duration of recordings, conscious mice	Invasive (surgical implantation of electrodes), external device connected and not always well tolerated, single housing
Implanted ECG telemetry systems	Long-term continuous 24/7 recordings, conscious mice in their natural living environment, simultaneous monitoring of other parameters	High cost, invasive (surgical implantation of transmitter), recovery period needed after implantation

Regardless of whatever technique is used–non-invasive or invasive–there are some differences between the mouse and human ECG which should be considered during analysis—in order to properly translate the insights gained from murine models to human conditions (Boukens et al. 2014; Kaese and Verheule 2012).

## Conclusion

Telemetry is a highly versatile tool for continuous long-term acquisition of ECG data in conscious freely moving mice in their home cage environment. It allows acquiring data 24/7 during different activities and can be combined with challenges. Most telemetry systems provide simultaneous monitoring of other physiological parameters, which is adding relevant information for ECG interpretation. However, telemetry transmitters require surgical implantation, the equipment for data acquisition is relatively expensive and analysis of the vast amount of data is challenging and time-consuming. Therefore, we suggest determining carefully if the use of telemetry is supposed to add valuable additional information to a study compared to the use of non-invasive methods. Primary screening with a non-invasive (and preferably non-restraining) method is easy and fast to perform and can give a first hint, however, subtle cardiac phenotypes might be masked or compensated due to stress during these procedures and thus remain undetected.

## Data Availability

Data sharing is not applicable to this article as no new data were created or analyzed in this review.
